# Green stability-indicating RP-HPLC method for simultaneous bioanalysis of tedizolid phosphate and ciprofloxacin in human plasma

**DOI:** 10.1038/s41598-026-65086-3

**Published:** 2026-08-13

**Authors:** Gehad Abd El-Fatah, Amr M. Mahmoud, Alaa A. Ahmed-Anwar, Ahmed A. Farghali, Rehab Mahmoud, Mohamed E. M. Hassouna

**Affiliations:** 1 Chemistry Department, Faculty of Science, Beni-Suef University, Beni-Suef, 62514 Egypt; 2 Analytical Chemistry Department, Faculty of Pharmacy, Cairo University, El-Kasr El-Aini Street, Cairo, 11562 Egypt; 3 Central Research Laboratory, Analytical Chemistry Department, Nahda University, Beni-Suef, Egypt; 4 Materials Science and Nanotechnology Department, Faculty of Postgraduate Studies for Advanced Sciences, Beni-Suef University, Beni-Suef, 62511 Egypt

**Keywords:** Tedizolid phosphate, Ciprofloxacin, RP-HPLC, Human plasma, Forced degradation, Greenness assessment, Biological techniques, Biotechnology, Chemistry, Environmental sciences

## Abstract

Tedizolid phosphate (TP) and ciprofloxacin (CIP) represent orally effective antibiotics that are often administered in combination for hospitalized patients with complicated or mixed infections, aiming to provide wide antimicrobial coverage. This study describes, for the first time, the establishment of an environmentally sustainable reversed-phase HPLC approach for the simultaneous bioanalysis of TP and CIP in human plasma. The developed chromatographic method allowed for rapid separation within 4 min employing a methanol–water mobile phase, thereby reducing solvent consumption and improving environmental sustainability. The method was validated according to the validation parameters investigated in the present study, with reference to the US FDA Bioanalytical Method Validation Guidance. Excellent linearity was achieved over concentration ranges of 5–90 µg/mL (TP) and 5–80 µg/mL (CIP) in pure samples, and 0.5–30 µg/mL (TP) and 0.5–10 µg/mL (CIP) in human plasma. LOD/LOQ values were 1.60/4.86 µg/mL for TP and 1.58/4.78 µg/mL for CIP in pure samples, and 0.16/0.49 µg/mL for TP and 0.10/0.31 µg/mL for CIP in spiked human plasma. The proposed method was characterized by adequate sensitivity and demonstrated strong performance in terms of accuracy, precision, specificity, and robustness. Furthermore, it proved effective in assessing the forced degradation of TP and CIP under diverse stress conditions, confirming its capability as a stability-indicating approach. The environmental impact of the method was comprehensively evaluated using multiple established greenness and blueness assessment tools, confirming its favorable environmental profile. Overall, the developed approach presents a rapid, highly accurate, and environmentally sustainable strategy for the simultaneous bioanalysis of TP and CIP in bulk drugs, pharmaceutical formulations, and human plasma.

**Supplementary Information:**

The online version contains supplementary material available at 10.1038/s41598-026-65086-3.

## Introduction

**Scheme 1:**
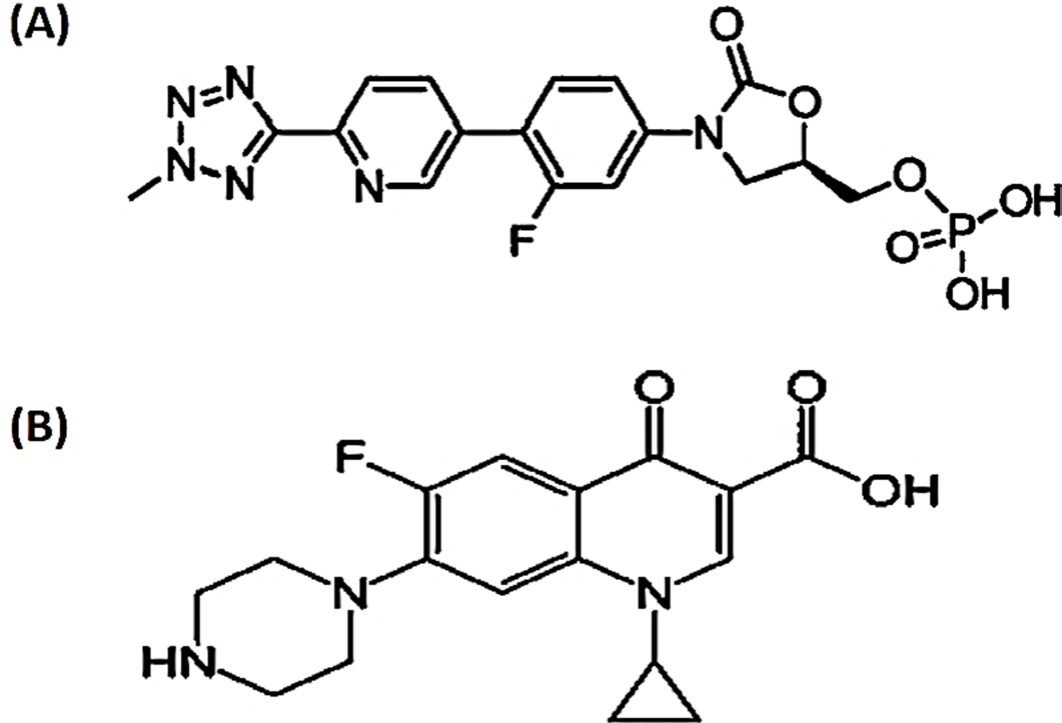
Chemical structure of tedizolid phosphate (**A**) and ciprofloxacin (**B**).

Tedizolid phosphate is chemically defined as [(5R)-(3-{3-fluoro-4-[6-(2-methyl-2 H-tetrazol-5-yl)pyridin-3-yl]phenyl}-2-oxooxazolidin-5-yl)methyl hydrogen phosphate] (Scheme 1A)^1^. It is characterized by an chemical structure of C₁₇H₁₆FN₆O₆P and a molecular weight of 450.32 g mol⁻¹^2,3^. Tedizolid phosphate (TP) is a prodrug that is rapidly converted into its active moiety, tedizolid, through enzymatic hydrolysis mediated by phosphatases. It is categorized as a second-generation oxazolidinone antibiotic, exerting its antibacterial activity by preventing protein synthesis via binding to the 50 S subunit of the bacterial ribosome^4,5^. In addition, TP exhibits strong antimicrobial efficacy against methicillin-resistant Staphylococcus aureus (MRSA), Mycobacterium tuberculosis, and various Gram-positive organisms, including those responsible for eye infections^6–8^. Ciprofloxacin is chemically defined as 1-cyclopropyl-6-fluoro-4-oxo-7-(piperazin-1-yl)-1,4-dihydroquinoline-3-carboxylic acid (Scheme 1B)^9^. It is characterized by an empirical formula of C₁₇H₁₈FN₃O₃ and a molecular weight of 331.34 g mol⁻¹^10,11^. Ciprofloxacin (CIP) is a second-generation fluoroquinolone antibiotic with broad therapeutic applications in the treatment of infections affecting the skin, soft tissues, gastrointestinal tract, bones, respiratory system, and urinary tract^12–16^. In clinical practice, particularly among hospitalized or critically ill patients with severe polymicrobial infections, empirical combination antimicrobial therapy is frequently initiated to provide broad-spectrum coverage while awaiting microbiological identification and antimicrobial susceptibility results^17–20^. Such treatment strategies are commonly employed in intensive care units, complicated skin and soft tissue infections, diabetic foot infections, intra-abdominal infections, and other healthcare-associated infections where both Gram-positive and Gram-negative pathogens may coexist^18–21^.

Tedizolid phosphate (TP) provides potent activity against Gram-positive organisms, including methicillin-resistant *Staphylococcus aureus* (MRSA), whereas ciprofloxacin (CIP) exhibits broad activity against many Gram-negative pathogens and selected Gram-positive bacteria^16,22–28^. Although TP and CIP are not routinely prescribed as a fixed therapeutic combination for a single specific indication, they may be co-administered as part of individualized treatment regimens in hospitalized patients requiring broad-spectrum antimicrobial coverage. Furthermore, previous studies have demonstrated the pharmaceutical compatibility of TP and CIP, supporting their concurrent administration when clinically indicated^29,30^. Therefore, the simultaneous determination of TP and CIP in biological matrices is clinically valuable for bioanalytical studies, clinical research, and quality control applications involving concurrent drug administration. High-performance liquid chromatography (HPLC) methods represent the most commonly employed analytical procedures and are regarded as highly important in the field of pharmaceutical analysis^31,32^. HPLC is characterized by multiple advantages such as wide applicability, reliable quantitative analysis, standardized procedures, reduced sample size requirements, and excellent resolution, accuracy, and sensitivity^33,34^. Various HPLC-based methods have been applied for the separate determination of TP in biological samples^2,6,35,36^. In the same context, numerous HPLC approaches have been established for the detection of CIP in biological matrices^37,38,47,39–46^. Consequently, a novel green RP-HPLC method is established for the simultaneous bioanalysis of TP and CIP in human plasma. In addition, an ICH-guided forced degradation study was performed to investigate the stability of the drugs under various stress conditions and to demonstrate the stability-indicating capability of the proposed method for quality control applications. A comprehensive evaluation employing the National Environmental Methods Index (NEMI), Analytical Greenness Profile (AGP), and Modified Green Analytical Procedure Index (Modified GAPI) methodologies was carried out to quantitatively evaluate the total sustainability and environmental footprint of the proposed method. The greenness of analytical chemistry is defined by reduced solvent consumption, the use of less toxic reagents, shorter analysis times, enhanced automation and integration, lower energy demand, and improved operational simplicity of analytical techniques^48^. Metrics serve as tools for evaluating performance enhancement, environmental impact, and the efficiency of chemical methodologies^49^. More recently, several green assessment approaches have been proposed to determine the compliance of analytical methods with green analytical chemistry principles. The NEMI represents a qualitative evaluation tool. Analytical GREEnness Metric Approach (AGREE)^50^ is a quantitative green assessment tool derived from the analytical Eco-scale approach, whereas GAPI^51,52^ is considered a semi-quantitative evaluation method. Recently, Blue Applicability Grade Index (BAGI) has been introduced as a complementary metric to assess the “blueness” of analytical methods, reflecting their practicality and applicability in real-world applications.

A comprehensive literature review reveals that, to the best of the authors’ knowledge, no HPLC method has been previously reported for the simultaneous bioanalysis of TP and CIP. Accordingly, this study presents a novel RP-HPLC method that is accurate, precise, sensitive, rapid, and environmentally sustainable for the simultaneous separation and quantification of TP and CIP in human plasma.

## Materials and methods

### Drugs and reagents

Tedizolid phosphate was obtained from Marcyrl Pharmaceutical Company (Cairo, Egypt) with a purity of 99.74 ± 0.94%, while ciprofloxacin was supplied by AMOUN Pharmaceuticals (Egypt) with a purity exceeding 99%. The pharmaceutical dosage forms were purchased from the local market, including Zolidocyrl tablets (Marcyrl Pharmaceutical Industries, El-Obour City, Egypt; each film-coated tablet contains 200 mg of TP) and ciprofloxacin tablets (Organo for Pharmaceutical and Chemical Industries, El-Obour City, Egypt; each film-coated tablet contains 500 mg of CIP). HPLC-grade methanol was obtained from Fisher Chemical. The mobile phase consisted of deionized water and methanol in a 60:40 (v/v) ratio, with the pH adjusted to 3.0 using orthophosphoric acid. Chromatographic separation was performed at a flow rate of 2.0 mL/min under ambient temperature (25 ± 0.5 °C), and UV detection was performed at 270 nm for the analysis of pure drugs, pharmaceutical formulations, and human plasma samples.

### Instruments

The chromatographic separation of the studied drugs was carried out using a Dionex Ultimate 3000 HPLC system (Massachusetts, USA) equipped with a quaternary solvent delivery pump, a diode array detector, and an autosampler. An X-Bridge BEH C18 column (5 μm, 4.6 × 250 mm) was used for separation. The mobile phase pH was adjusted using a Jenway pH meter (Model 3510, Staffordshire, UK).

### Standard stock and working solutions

Stock solutions of TP and CIP (100 µg/mL) were prepared by accurately weighing 10 mg of each drug and dissolving it in a 100 mL volumetric flask using a methanol–deionized water mixture (55:45, v/v). The solutions were stirred for 30 min, cooled to room temperature, and stored at 4 °C until analysis. Working standard solutions were prepared by appropriate dilution of the stock solutions with methanol to obtain final concentrations ranging from 5 to 90 µg/mL for TP and 5–80 µg/mL for CIP. All prepared solutions were filtered through syringe membrane filters prior to injection.

### Human plasma samples

Blank human plasma was obtained from one healthy, non-smoking volunteer after an 8-hour fasting period with no intake of food or water. Blood was collected into tubes containing ethylenediaminetetraacetic acid (EDTA) as an anticoagulant and centrifuged at 3000 rpm for 15 min. The separated plasma was stored at 4 °C until analysis. Individual plasma was used throughout the study. In this study plasma sample collection was conducted in accordance with the ethical approval obtained from the Ethics Committee of the Faculty of Postgraduate Studies for Sciences, Beni-Suef University (Approval No.: PSAS-BSU-HAREC.004), in compliance with the applicable ethical standards.

### Calibration samples

Calibration standards were prepared by accurately transferring varying aliquots of TP and CIP stock solutions (100 µg/mL) into 10 mL volumetric flasks, followed by dilution to volume with methanol. This procedure produced calibration concentrations ranging from 5 to 90 µg/mL for TP and 5–80 µg/mL for CIP in pure samples.

Calibration samples in plasma were prepared by spiking appropriate aliquots of TP and CIP stock solutions (100 µg/mL) into blank human plasma. Subsequently, 300 µL of salicylic acid working solution (1000 µg/mL) was added to each sample as the internal standard prior to protein precipitation. The same volume and concentration of the internal standard were used for all calibration standards, quality control samples, and plasma samples throughout the study to ensure consistent quantification.

This procedure produced calibration concentrations ranging from 0.5 to 30 µg/mL for TP and 0.5–10 µg/mL for CIP in human plasma.

### Pharmaceutical formulation stock solutions

Ten tablets of each formulation (Zolidocyrl^®^ 200 mg TP/tablet and Ciprofloxacin 500 mg CIP/tablet) were weighed and finely powdered. The average tablet weight was calculated, and an accurately weighed portion equivalent to 10 mg of each drug was dissolved in a methanol–deionized water mixture (55:45, v/v) using sonication for 30 min. The solution was then cooled to room temperature, transferred into a 100 mL volumetric flask, and filtered through a syringe membrane filter prior to analysis. Additional dilutions were prepared by transferring suitable aliquots of the stock solution into 10 mL volumetric flasks and diluting to volume with methanol to obtain final concentrations of 5, 10, and 20 µg/mL for both TP and CIP prior to HPLC analysis. The drug contents of the pharmaceutical formulations were then determined using the corresponding calibration curves.

### Stress degradation conditions

TP and CIP were investigated under multiple stress conditions to determine their susceptibility to degradation during storage and use. Such forced degradation can occur through acidic, alkaline, oxidative, neutral, photolytic, and thermal mechanisms. For the forced degradation study, tablet samples were prepared at a concentration of 50 µg/mL to ensure adequate detector response and reliable monitoring of degradation products under stress conditions. This concentration was selected specifically for stress testing and was independent of the concentration range used for routine quantitative analysis. A stock solution of the pharmaceutical tablet formulation containing TP and CIP (100 µg/mL), expressed as the labeled drug content) was prepared as described in the Pharmaceutical Formulation section. This stock solution was used throughout all forced degradation experiments.

### Acidic Degradation

A 2.5 mL aliquot of the TP and CIP tablet stock solution (100 µg/mL) was combined with 2.5 mL of 0.01 M HCl to obtain a final concentration of 50 µg/mL. The mixture was divided into two portions, one stored at room temperature and the other heated at 80 °C, and both were analyzed after 1 and 2 h.

### Alkaline Degradation

The procedure employed for acid hydrolysis was similarly followed for alkaline degradation. A 2.5 mL aliquot of the TP and CIP tablet stock solution (100 µg/mL) was mixed with 2.5 mL of 0.01 M NaOH to obtain a final concentration of 50 µg/mL. The resulting solution was split into two portions, one maintained at room temperature and the other heated at 80 °C, and analyzed after 1 and 2 h.

### Oxidative Degradation

Oxidative degradation was performed in the absence of light by mixing 2.5 mL aliquot of the TP and CIP tablet stock solution (100 µg/mL) with 2.5 mL of 5% hydrogen peroxide, resulting in a final concentration of 50 µg/mL. The solution was split into two portions, one kept at room temperature and the other incubated at 80 °C, and analyzed after 1 and 2 h.

### Neutral Degradation

Distilled water was used to dilute 2.5 mL aliquot of the TP and CIP tablet stock solution (100 µg/mL) to a final volume of 5 mL, resulting in a concentration of 50 µg/mL. The solution was then incubated in a water bath at 80 °C and analyzed after 1 and 2 h.

### Photolytic Degradation

Samples of TP and CIP tablet stock solution (100 µg/mL) were exposed to sunlight, and analysis was carried out after 1 and 2 h.

### Thermal Degradation

Thermal stress conditions were applied by heating aliquots of TP and CIP tablet stock solution (100 µg/mL) in a water bath at 80 °C for 2 h.

## Results and discussion

### RP-HPLC assay optimization

#### Selecting the suitable internal standard (IS)

Several compounds, including Mirabegron, Ibuprofen, Phenylephrine HCl, Streptomycin sulfate, Methocarbamol, Hydrocortisone, Solifenacin succinate, Guaifenesin, and Salicylic acid, were experimentally evaluated as potential internal standards. Among them, salicylic acid exhibited the most favorable chromatographic performance, producing a sharp and well-resolved peak without interference from tedizolid phosphate, ciprofloxacin, or endogenous plasma components. Throughout method validation and plasma sample analysis, salicylic acid consistently exhibited a stable and reproducible chromatographic response, while its retention time remained consistent under the optimized chromatographic conditions, confirming its suitability as the internal standard for the proposed bioanalytical method. Based on these findings, salicylic acid was selected as the most suitable internal standard for the proposed method.

#### Selecting a suitable solvent for protein precipitation

Protein precipitation was investigated using methanol and acetonitrile. Methanol provided the cleanest chromatograms with minimal endogenous plasma interference and good peak symmetry for both TP and CIP. Therefore, methanol was selected for subsequent plasma sample preparation. After the addition of 2.0 mL methanol, the samples were centrifuged at 3000 rpm for 15 min to ensure efficient protein removal. The collected supernatant was evaporated to dryness in a water bath maintained at 70 °C to remove methanol used for protein precipitation. The residue was subsequently reconstituted with a deionized water–methanol mixture adjusted to pH 3.0 with orthophosphoric acid to provide a solvent compatible with the mobile phase, thereby minimizing solvent effects and improving chromatographic performance. The samples were then analyzed using reverse-phase HPLC on an X-Bridge BEH C18 column (5 μm, 4.6 × 250 mm).

#### Optimization of the mobile phase and flow rate ratio

**Fig. 1:**
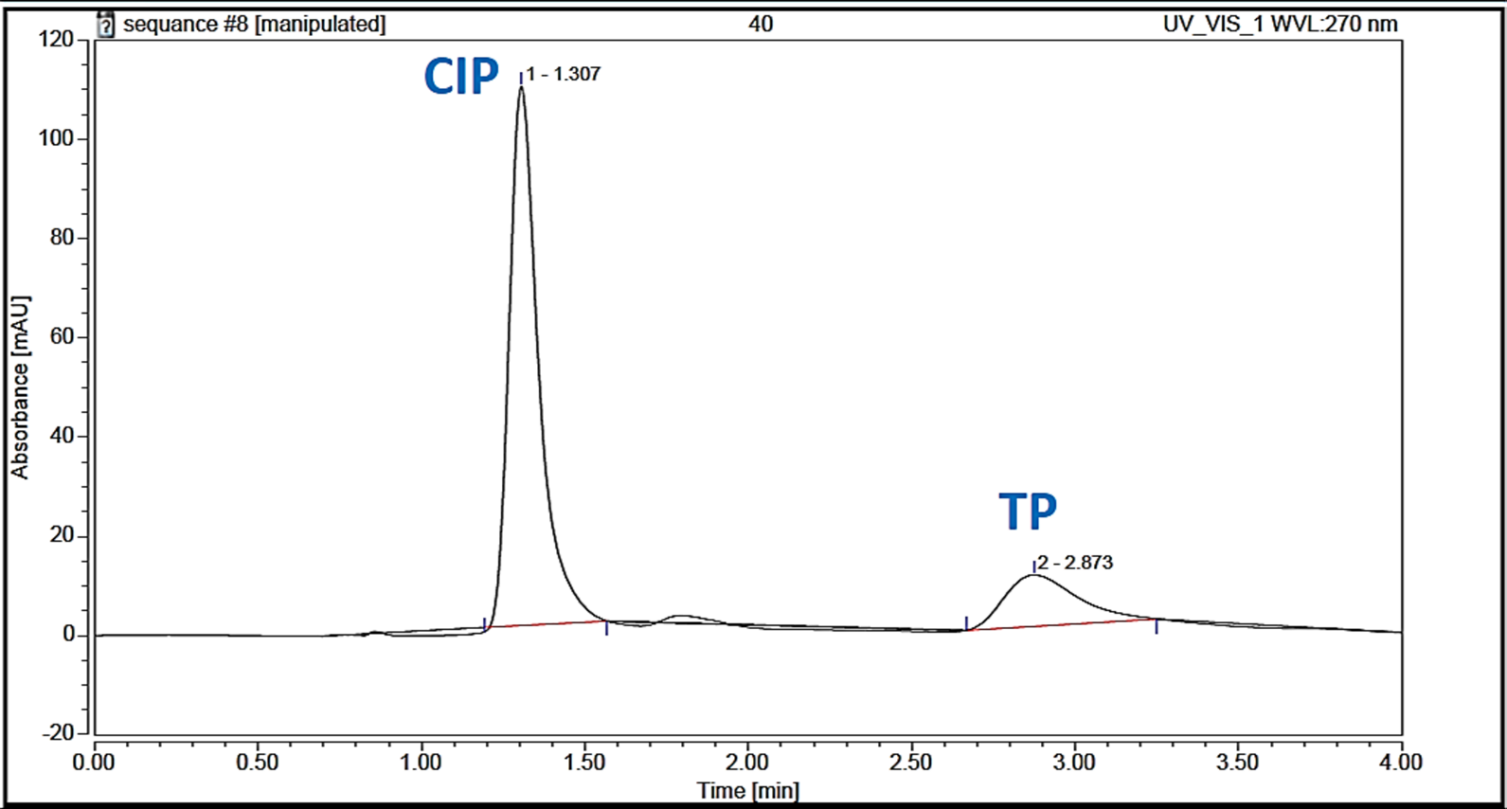
RP-HPLC chromatogram of TP and CIP separation in pure samples, performed at a flow rate of 2.0 mL/min for 4.0 min and monitored at a wavelength of 270 nm.

**Fig. 2:**
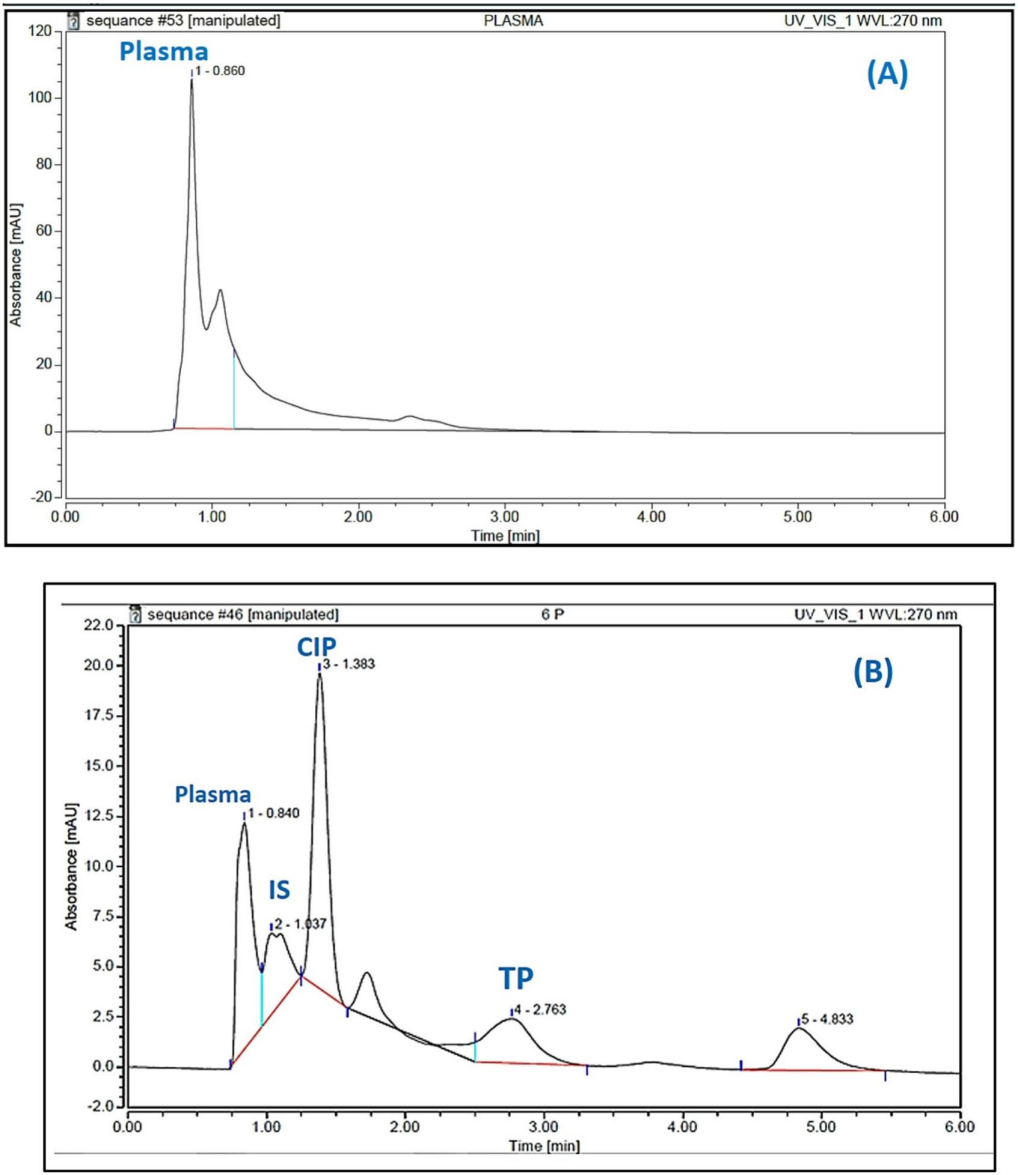
RP-HPLC chromatograms of human plasma **(A)** and TP–CIP separation in human plasma with IS **(B)**, recorded at a wavelength of 270 nm.

Several experimental runs were performed to achieve effective separation of TP and CIP by modifying both the mobile phase ratio and the flow rate. Different proportions of deionized water and methanol (50:50 to 30:70) were assessed, with flow rates set between 1.0 and 2.0 mL/min. It was found that reducing the pH enhanced the peak sharpness of CIP. The effect of pH was studied across values from 3.0 to 9.0, and CIP showed optimal peak separation at pH 3.0. Based on multiple separation trials, the optimized mobile phase consisted of 60% deionized water and 40% methanol adjusted to pH 3.0 by orthophosphoric acid, applied at a flow rate of 2.0 mL/min for a total run time of 4.0 min. Additionally, the separation of TP and CIP in both pure samples and plasma was performed at a wavelength of 270 nm. Figures 1 and 2 illustrate the successful and complete separation of the TP and CIP mixture in pure samples and human plasma following optimization of the chromatographic conditions.

### Construction of Calibration Curves

Two separate calibration curves for TP and CIP were prepared at 270 nm, one for pure samples and another for human plasma. Calibration utilized TP–CIP mixtures at a fixed 1:1 (w/w) proportion. Each sample (20 µL) was injected and analyzed in triplicate using the optimized RP-HPLC method. Chromatographic separation was performed using a mobile phase of 60% deionized water and 40% methanol adjusted to pH 3.0 by orthophosphoric acid, at a flow rate of 2.0 mL/min and a run time of 4.0 min. Calibration curves for pure samples were obtained by plotting the average peak area vs. the corresponding concentrations, leading to the derivation of regression equations. Regarding the regression results, the limits of detection (LOD) and quantification (LOQ) were calculated. Table 1 lists the working concentrations and associated peak areas of TP and CIP in pure samples. In the case of human plasma, TP and CIP peak responses were expressed relative to the internal standard. Regression equations were established by plotting the drug-to-internal standard peak area ratio against the corresponding concentrations. Table 2 provides the working solution concentrations and associated peak areas of TP and CIP in plasma samples. A fresh calibration curve was prepared and analyzed for each analytical run using newly prepared calibration standards covering the validated concentration range. The concentrations of the analyzed samples were calculated using the corresponding calibration curve generated for that specific analytical run.

**Table 1:** Calibration data for TP and CIP in pure samples.

Tedizolid phosphate (TP)		Ciprofloxacin (CIP)	
**Concentration (µg/mL)**	**Peak area (mAU·min)**	**Concentration (µg/mL)**	**Peak area (mAU·min)**
5	0.084	5	0.247
10	0.197	25	4.918
25	1.208	30	6.284
40	2.340	40	9.050
50	2.992	60	14.129
60	3.590	70	16.317
90	5.768	80	18.707

**Table 2:** Series of measured working solution concentrations and their corresponding peak areas for TP and CIP in human plasma.

Tedizolid phosphate (TP)		Ciprofloxacin (CIP)	
**Concentration (µg/mL)**	**Peak area (mAU·min)**	**Concentration (µg/mL)**	**Peak area (mAU·min)**
0.5	0.004	0.5	0.054
1	0.018	1	0.171
2	0.068	2	0.356
6	0.317	4	0.785
8	0.453	6	1.254
10	0.532	8	1.703
20	1.166	10	2.151
30	1.798		

### Greenness profile assessment

The environmental friendliness of analytical methodologies is governed by factors such as reagent consumption and hazard, waste production, process steps, energy requirements, automation, and miniaturization. A systematic evaluation using NEMI, AGP, and Modified GAPI was conducted to determine the greenness of the proposed approach. The Analytical Eco-scale and AGREE metrics were used as green evaluation tools. The Eco-scale score is derived from a baseline of 100, with deductions applied for energy requirements, chemical use, waste production, and toxicity^53^. Another approach for evaluating greenness is AGREE, a rapid scoring system that quantitatively reflects adherence to the 12 principles of Green Analytical Chemistry^54^. The BAGI metric, recently introduced, measures an analytical method’s “blueness,” indicating both efficiency and applicability^55^. It offers a quantitative evaluation across ten parameters, such as analysis type, analyte number, instrument requirements, sample preparation, throughput, reagent consumption, preconcentration necessity, sample volume, and automation capabilities.

### Procedure validation

The proposed analytical procedure was validated according to the validation parameters investigated in this study, including linearity, sensitivity (LOD and LOQ), precision, accuracy, specificity, robustness, and stability, with reference to the US FDA Bioanalytical Method Validation Guidance^56^. For plasma validation, three quality control (QC) levels were selected within the validated calibration range: low-quality control (LQC, 0.5 µg/mL), medium-quality control (MQC, 2 µg/mL), and high-quality control (HQC, 10 µg/mL). These QC levels were consistently used for the evaluation of precision, accuracy, and stability. According to the US FDA Bioanalytical Method Validation Guidance, precision was considered acceptable when the %CV (or %RSD) did not exceed 15% for QC samples (LQC, MQC, and HQC) and 20% at the lower limit of quantification (LLOQ).

### Linearity and ranges

The linearity of tedizolid phosphate (TP) and ciprofloxacin (CIP) was established over concentration ranges of 5–90 µg/mL and 5–80 µg/mL for pure samples, respectively. In human plasma, the linear ranges were 0.5–30 µg/mL for TP and 0.5–10 µg/mL for CIP.

Each concentration level was injected in triplicate, and linear calibration curves were constructed by plotting peak area versus concentration using linear regression analysis. The calibration curves for pure samples showed regression coefficients of 0.9990, whereas plasma samples demonstrated slightly higher values (Table 3). As illustrated in Figs. S1A–B and S2A–B, peak area exhibited a clear linear relationship with drug concentration in both matrices.

The back-calculated concentrations together with the corresponding %Bias and %RSD values for all plasma calibration standards are provided in the Supplementary Information (Tables S1 and S2).

### Limit of detection (LOD) and limit of quantitation (LOQ)

The LOD and LOQ values were calculated according to the ICH Q2(R1) guideline using the calibration curve slope (S) and the standard deviation of the response (σ), according to the following equations:

1 $$\:\mathrm{L}\mathrm{O}\mathrm{D}\hspace{0.17em}=\hspace{0.17em}3.3\times\:{\upsigma\:}/\mathrm{S}$$

2 $$\:\mathrm{L}\mathrm{O}\mathrm{Q}\hspace{0.17em}=\hspace{0.17em}10\times\:{\upsigma\:}/\mathrm{S}$$

In pure samples, LOD and LOQ values were 1.60 and 4.86 µg/mL for TP and 1.58 and 4.78 µg/mL for CIP, respectively. In spiked plasma, these values were reduced to 0.16 and 0.49 µg/mL for TP and 0.10 and 0.31 µg/mL for CIP, respectively. As shown in Table 4, the current study is compared with earlier reported methods for TP and CIP analysis. The proposed method offers improved sensitivity, better resolution, and reduced analysis time, making it more efficient for routine pharmaceutical applications.

**Table 3:** Validation parameters of the proposed RP-HPLC method for TP and CIP in pure samples and human plasma at 270 nm.

Parameters	Pure samples TP	Pure samples CIP	Plasma TP	Plasma CIP
**Range (µg/mL)**	5–90	5–80	0.5–30	0.5–10
**Slope**	0.0678	0.2490	0.0610	0.2211
**Intercept**	-0.4038	-1.0827	-0.0469	-0.0699
**Correlation coefficient (R** ^**2**^ **)**	0.9990	0.9990	0.9992	0.9994
**LOD (µg/mL)**	1.60	1.58	0.16	0.10
**LOQ (µg/mL)**	4.86	4.78	0.49	0.31

**Table 4:** Comparison between previous studies and the newly developed RP-HPLC method for the separation and determination of TP and CIP.

TP data						
Method and Mobile Phase	LOD (µg/mL)	LOQ (µg/mL)	Retention Time (min)	Wavelength (nm)	Sample Type	Reference
HPLC/Mixture of aqueous buffer of pH 7.0 of disodium hydrogen phosphate with additive ß-cyclodextrin, triethylamine, and acetonitrile	0.1	0.3	0.93	300	Pure samples	^2^
HPLC/ Phosphate buffer (pH 6.5):acetonitrile in the ratio of 70:30 (%v/v)	0.25	1.0	2.82	300	Pharmaceutical formulations	^35^
HPLC/ Sodium acetate buffer (pH adjusted to 3.5 by Hydrochloric acid) and acetonitrile in the ratio of 65:35 (%v/v)	< 0.25	0.25>	The total run time was 10 min	251	Pure samples	^6^
HPLC/ Phosphate buffer and acetonitrile in the ratio of 80:20 (%v/v)	0.57772	1.75067	Not specified	241	Pharmaceutical Dosage Form	^57^
RP-HPLC/ DI : Methanol adjusted by ortho-phosphoric acid to pH 3.0 in the ratio of 60:40 (%v/v)	0.16	0.49	2.76	270	Human plasma	**Current study**
**CIP data**						
**Method and Mobile Phase**	**LOD (µg/mL)**	**LOQ (µg/mL)**	**Retention time (min)**	**Wavelength (nm)**	**Sample Type**	**Reference**
HPLC/Methanol: 0.25 mM potassium phosphate buffer in the ration of 60:40 (%v/v)	0.33	0.99	10	282	Bulk and Pharmaceutical Formulation	^58^
HPLC/Acetonitrile: water adjusted to pH 3.0 with diluted acetic in the ratio of 65:35 (% v/v)	0.33	0.44	2.75	210	Human plasma	^37^
HPLC / Methanol : Phosphate buffer (pH 3.9) in the ratio of 40:60 (%v/v)	0.1	5	6.1	279	Wastewater	^59^
HPLC/ Methanol : 0.1% aqueous formic acid) in the ratio of 70:30 (%v/v)	0.132	0.401	2.71	Not specified	Pharmaceutical formulations	^60^
HPLC/ 0.025 M phosphoric acid (adjusted to pH 3.0 ± 0.1 with triethanolamine) and acetonitrile in the ratio of 60:40 (%v/v)	0.11	0.35	Not specified	278	Pharmaceutical preparations	^9^
HPLC/Methanol: 0.018 M phosphate buffer (pH 3.0) in the ratio of 59:41 (%v/v)	0.169	0.573	3.522	270	Pharmaceutical formulations, human serum and urine	^61^
RP-HPLC/ DI : Methanol adjusted by ortho-phosphoric acid to pH 3.0 in the ratio of 60:40 (%v/v)	0.10	0.31	1.38	270	Human plasma	**Current study**

### Precision and accuracy

Analytical precision and accuracy were evaluated at three QC levels (LQC, MQC, and HQC). For precision assessment, intraday and interday analyses were performed in triplicate at each QC level. Concentrations were calculated using the corresponding regression equations, and %RSD and recovery values were determined accordingly. The data presented in Table 5 demonstrate that %RSD values were consistently below 1.0%, indicating excellent precision of the developed method. The RP-HPLC method also demonstrated good accuracy for the quantification of TP and CIP in spiked human plasma samples. The analytical recovery values ranged from 98.0% to 100.5%, while the corresponding %bias values ranged from − 3.10% to + 0.50%, and the mean recovery values were within the established acceptance limits (Table 6), confirming the reliability of the proposed method.

**Table 5:** Precision results (intra-day and inter-day) of the suggested analytical method in human plasma at LQC (0.5 µg/mL), MQC (2 µg/mL), and HQC (10 µg/mL).

Drug	Concentration (µg/mL)	Intra-day Recovery %	Intra-day RSD %	Inter-day Recovery %	Inter-day RSD %
**TP**	0.5 2 10	99.70 99.40 98.90	0.24 0.52 0.41	101.60 99.90 102.30	0.73 0.81 0.67
**CIP**	0.5 2 10	99.80 101.40 98.20	0.48 0.39 0.35	100.60 99.50 98.70	0.61 0.84 0.72

**Table 6:** Analytical recovery (accuracy) and %bias of tedizolid phosphate and ciprofloxacin in spiked human plasma samples.

Drug	Concentration (µg/mL)	Recovery %	%Bias	Mean Recovery ± SD
**TP**	0.5	98.40	−1.60	98.80 ± 1.72
	2	97.60	−2.40	
	10	100.50	+ 0.50	
**CIP**	0.5	99.20	−0.80	98.0 ± 1.84
	2	96.90	−3.10	
	10	97.80	−2.20	

### Specificity

As defined by ICH guidelines, specificity is the capability of an analytical procedure to accurately identify the analyte in the presence of other components, including impurities, degradation products, and matrix substances. The chromatographic profiles showed distinct and well-separated peaks, indicating the absence of interference and the method’s specificity. In addition, specificity was evaluated using processed blank human plasma obtained from a single donor. No endogenous interfering peaks were observed at the retention times of tedizolid phosphate, ciprofloxacin, or the internal standard.

### Carry-over assessment

Carry-over was evaluated by injecting a processed blank plasma sample immediately after the highest plasma calibration standard (30 µg/mL for TP). No detectable peaks were observed at the retention times of tedizolid phosphate, ciprofloxacin, or the internal standard in the subsequent blank chromatogram, indicating the absence of carry-over under the optimized chromatographic conditions, as illustrated in Figs. S3A and S3B.

### Dilution Integrity

Dilution integrity was evaluated using plasma samples spiked at concentrations above the validated calibration range. Tedizolid phosphate (TP) was evaluated at 60 µg/mL, while ciprofloxacin (CIP) was evaluated at 20 µg/mL. The samples were diluted two-fold with blank plasma prior to analysis. The diluted TP samples demonstrated acceptable accuracy and precision, with a mean measured concentration of 53.93 µg/mL, a bias of − 10.11%, and an RSD of 0.08%. Similarly, the diluted CIP samples showed a mean measured concentration of 17.11 µg/mL, a bias of − 14.43%, and an RSD of 0.02%. These results confirm that sample dilution did not adversely affect the analytical performance of the proposed method and complied with the acceptance criteria of the US FDA Bioanalytical Method Validation Guidance. The corresponding dilution integrity data are provided in the Supplementary Information (Table S3).

### Extraction recovery

Extraction recovery was evaluated at three quality control (QC) levels (LQC, MQC, and HQC) using methanol as the protein-precipitating solvent and plasma obtained from a single donor. The extraction recovery of tedizolid phosphate (TP) ranged from 97.74% to 98.10%, while that of ciprofloxacin (CIP) ranged from 96.01% to 97.52%, demonstrating efficient and consistent extraction of both analytes from spiked human plasma under the optimized experimental conditions. The detailed extraction recovery results are provided in the Supplementary Information (Table S4).

### Matrix effect

Matrix factor (MF) was calculated as the ratio of the analyte peak response in post-extraction spiked plasma to that of the corresponding neat standard at the same concentration, using three quality control (QC) levels (LQC, MQC, and HQC). Matrix factor (MF) values ranged from 0.970 to 1.067 for tedizolid Phosphate (TP) and from 1.001 to 1.050 for ciprofloxacin (CIP), indicating negligible matrix effect under the investigated experimental conditions. The detailed results are provided in Supplementary Information (Table S5). Since the assessment was performed using a single human plasma source rather than multiple plasma lots, the presented data represent a limited assessment of matrix effects.

### Robustness

The robustness of an analytical method is defined as its ability to withstand minor intentional changes in analytical conditions without significant impact on its performance, reflecting its reliability in normal application. It was assessed by examining system suitability solutions along with batch analyses performed under deliberately varied parameters. The method robustness was assessed by varying key parameters such as flow rate (2.0–2.4 mL/min), detection wavelength (270, 280, and 300 nm), and mobile phase composition (60:40 to 58:42% v/v of DI water and methanol). The results demonstrated %RSD values lower than 1.0%, confirming the robustness of the developed method.

### Stability

Drug stability in plasma samples was evaluated under routine analytical conditions, including bench-top stability at room temperature (25 ± 0.5 °C) and freeze–thaw stability (three cycles). As summarized in Table 7, TP and CIP were stable for 6 h at room temperature and 7 days under frozen storage conditions. The obtained recovery and %RSD values confirmed the stability of both analytes under the investigated conditions.

**Table 7:** Evaluation of drug stability in human plasma under different storage conditions.

Drug	Concentration (µg/mL)	Room temperature (25 ± 0.5 °C)		Freeze–thaw stability (three cycles)	
		Recovery %	Room temperature: RSD %	Recovery %	Freeze– thaw stability: RSD %
**TP**	0.5 2 10	96.30 98.70 94.50	1.06 0.64 0.88	99.40 100.50 101.30	0.64 0.42 0.71
**CIP**	0.5 2 10	95.80 102.70 93.90	0.76 0.88 1.13	99.70 101.40 98.50	0.51 0.68 0.37

### Analysis of pharmaceutical formulation

The commercial formulations containing TP and CIP were subjected to direct analysis of tablet solutions, allowing simultaneous determination of both drugs in a 1:1 mixture. Drug concentrations were calculated based on the regression equations, and recovery results are listed in Table 8. Recoveries were found to be in the range of 98.10–103.20% with low %RSD values, demonstrating the reliability of the method for routine analysis. The obtained results indicate that the developed method provides good accuracy for the concurrent quantification of tedizolid phosphate and ciprofloxacin in pharmaceutical preparations. The satisfactory recovery values and low %RSD further indicate that no significant interference from tablet excipients affected the chromatographic determination of the target analytes under the proposed chromatographic conditions.

**Table 8:** Assay results of ciprofloxacin and tedizolid phosphate in pharmaceutical formulations.

Pharmaceutical formulation	Taken (µg/mL)	TP Found	TP Recovery %	TP RSD %	CIP Found	CIP Recovery%	CIP RSD%
A mixed solution was formulated at an equal ratio (1:1) using Zolidocyrl (200 mg TP/tablet) and ciprofloxacin (500 mg CIP/tablet) pharmaceutical products.	5	5.16	103.20	0.56	4.96	99.27	0.18
	10	9.82	98.20	0.41	10.01	100.14	0.16
	20	19.70	98.50	0.35	19.62	98.10	0.23

### Stressed degradation study

**Fig. 3:**
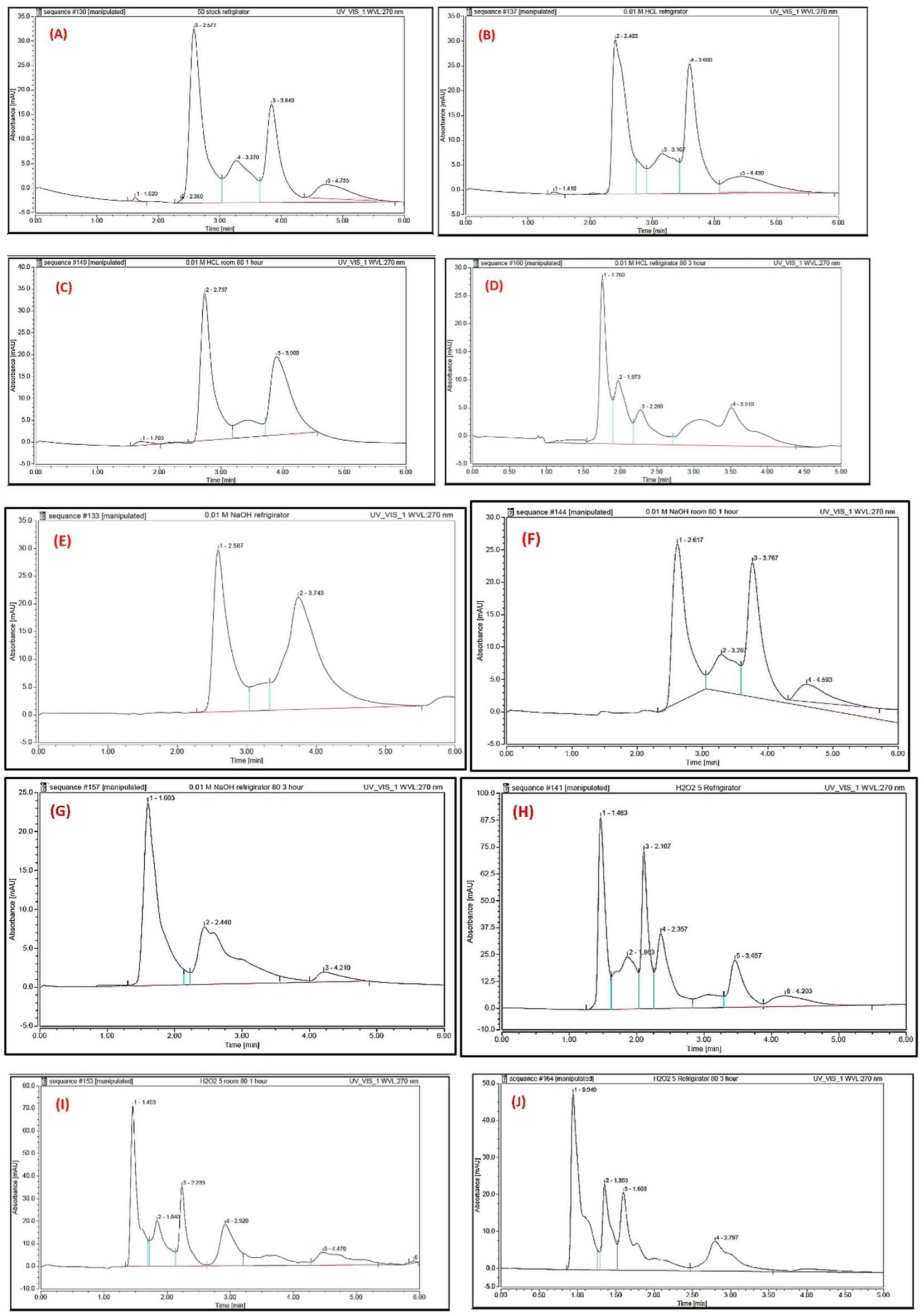

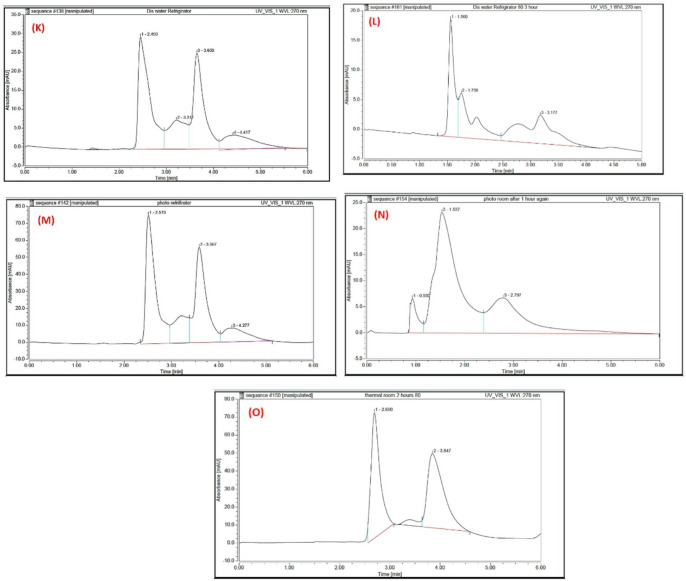
Chromatographic profiles TP and CIP in the pharmaceutical formulation under forced degradation conditions: **(A)** unstressed (control) conditions, **(B)** 0.01 M HCl at room temperature, **(C)** 0.01 M HCl at 80 °C for 1 h, **(D)** 0.01 M HCl at 80 °C for 2 h, **(E)** 0.01 M NaOH at room temperature, **(F)** 0.01 M NaOH at 80 °C for 1 h, **(G)** 0.01 M NaOH at 80 °C for 2 h, **(H)** 5% H_2_O_2_ at room temperature, **(I)** 5% H_2_O_2_ at 80 °C for 1 h, **(J)** 5% H_2_O_2_ at 80 °C for 2 h, **(K)** distilled water at 80 °C for 1 h, **(L)** distilled water at 80 °C for 2 h, **(M)** photolytic degradation after 1 h under sunlight, **(N)** photolytic degradation after 2 h under sunlight, and **(O)** thermal degradation at 80 °C for 2 h.

Figures 3 (A–O) illustrate the degradation profiles of TP and CIP, whereas Table 9 provides the corresponding percentage degradation values obtained from forced degradation studies employing the developed method. To investigate the stability characteristics of TP and CIP in the pharmaceutical formulation, forced degradation studies were performed under a range of stress conditions, such as acidic, alkaline, oxidative, neutral, photolytic, and thermal environments. The findings demonstrated that the extent of degradation for both drugs was highly dependent on the stress conditions applied. In acidic medium (0.01 M HCl), TP showed stability at ambient temperature and upon heating at 80 °C for 1 h; however, slight degradation (17.83%) occurred after 2 h. On the other hand, CIP demonstrated higher sensitivity to acidic hydrolysis, with degradation percentages reaching 12.63%, 15.88%, and 61.71% at room temperature, after 1 h, and after 2 h of thermal exposure, respectively. TP demonstrated satisfactory stability in alkaline medium (0.01 M NaOH) at room temperature and after 1 h of heating, whereas moderate degradation (25.60%) was observed after 2 h at 80 °C. In contrast, CIP exhibited greater susceptibility to alkaline degradation, with degradation percentages of 14.66% at room temperature, increasing to 30.67% after heating at 80 °C for 1 h and to 31.94% after 2 h, indicating the progressive effect of thermal alkaline stress. TP showed pronounced degradation under oxidative stress (5% H₂O₂) upon heating, reaching 23.15% and 21.76% after 1 and 2 h at 80 °C, respectively, while CIP exhibited lower susceptibility. Under neutral conditions, TP maintained stability after 1 h at 80 °C but displayed marked degradation (48.97%) following extended heating. CIP exhibited even higher susceptibility, with degradation reaching 74.25% after 2 h. Exposure to sunlight further revealed that both drugs are sensitive to photolytic degradation. A notable degradation of TP (47.51%) was observed after 2 h, in contrast to moderate degradation of CIP (32.33%). Under thermal conditions (80 °C for 2 h), TP was comparatively stable, while CIP showed moderate degradation (23.22%) In general, the results demonstrate that CIP is more susceptible to degradation across most stress conditions, while TP shows comparatively higher stability, with the exception of extended thermal and photolytic conditions.

**Table 9:** Percentage degradation of TP and CIP in the pharmaceutical formulation under various forced degradation conditions.

Stress testing with recommended conditions		TP Degradation %	CIP Degradation %
**Unstressed (control) conditions**		0.00	0.00
**Acid Degradation** **(0.01 M HCl)**	At room temperature At 80 °C for 1 h At 80 °C for 2 h	0.00 0.00 17.83	12.63 15.88 61.71
**Base Degradation** **(0.01 M NaOH)**	At room temperature At 80 °C for 1 h At 80 °C for 2 h	0.00 0.00 25.60	14.66 30.67 31.94
**Oxidative Degradation** **(5% H**_**2**_ **O**_**2**_ **)**	At room temperature At 80 °C for 1 h At 80 °C for 2 h	0.00 23.15 21.76	0.00 2.76 22.15
**Neutral Degradation**	At 80 °C for 1 h At 80 °C for 2 h	0.00 48.97	7.92 74.25
**Photolytic Degradation**	After 1 h under sunlight After 2 h under sunlight	0.00 47.51	3.42 32.33
**Thermal Degradation** (At 80 °C for 2 h)		0.00	23.22

### Greenness assessment of the applied methods

A single assessment method is insufficient to capture all dimensions of sustainability^62^. Green Analytical Chemistry (GAC) is an analytical framework focused on reducing or avoiding solvents, reagents, and other hazardous components that may negatively impact the environment and human health. GAC provides a balance between strong analytical performance, rapid processing, and reduced costs^63^. The environmental consequences and sustainability of analytical procedures should be evaluated to clarify their impact on ecosystems. The concept of “sustainability” broadly covers affordability, analytical efficiency, waste minimization, safety, and method greenness. In this work, the environmental friendliness of the developed approach was assessed using NEMI, AGP, Modified GAPI, Analytical Eco-Scale, AGREE, and BAGI providing a comprehensive multi-metric evaluation of the sustainability of the proposed analytical method.The greenness assessment was performed exclusively for the proposed RP-HPLC analytical method under the optimized routine analytical conditions. The reagents used for the forced degradation studies were not included in the greenness evaluation because these experiments were conducted solely to demonstrate the stability-indicating capability of the method and do not constitute part of the routine analytical procedure.

### The NEMI greenness assessment method

**Fig. 4:**
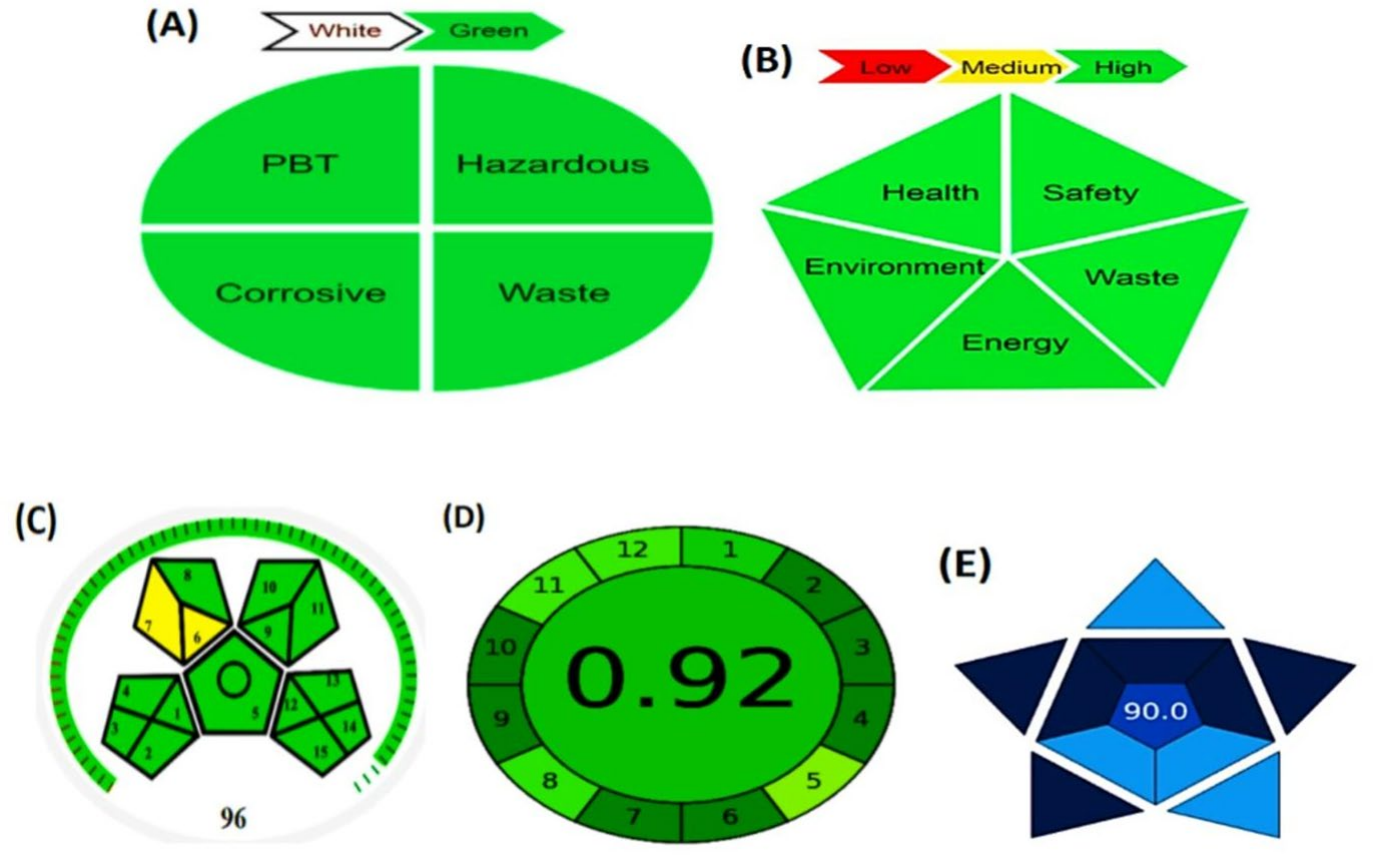
**(A)** Pictorial illustration of the NEMI greenness metric; **(B)** pictorial illustration of the AGP greenness metric; **(C)** pictogram of the modified GAPI tool; **(D)** aggregated AGREE score for the developed analytical method; **(E)** BAGI assessment of method blueness.

The NEMI was established in 2002 by the Methods and Data Comparability Board (MDCB)^54^, represents one of the first evaluation tools in green analytical chemistry and serves as a searchable database. The NEMI pictogram consists of a circular diagram divided into four parts, with each section representing a defined requirement. If the criterion is met, the corresponding segment is highlighted in green. If the requirement is not satisfied, the respective segment remains uncolored. The initial section of the NEMI pictogram is marked in green when none of the reagents used in the analytical procedure are classified as persistent, bioaccumulative, and toxic (PBT) chemicals. The second segment of the NEMI pictogram is colored green when the solvents used in the analytical procedure are non-hazardous and are not classified under D, F, P, or U waste categories. The third portion of the NEMI pictogram is highlighted in green when the sample pH falls within the 2–12 range, thereby limiting adverse environmental impacts. The fourth portion of the NEMI pictogram is highlighted in green when the generated waste is ≤ 50 g. As one of the first greenness assessment approaches, NEMI presents several benefits^64^. Moreover, a simple look at the NEMI pictogram gives a rapid and general indication of the environmental impact of the analytical procedures, as presented in Fig. 4A.

### The AGP greenness assessment method

The NEMI framework was further improved and transformed into the AGP metric. Compared with the original NEMI system, AGP includes five segments that evaluate method greenness in relation to safety, energy, human health, waste, and environmental impact. Each component’s greenness is determined using National Fire Protection Association (NFPA) scores and specified dosage limits, and is illustrated in the pictogram through three color indicators. Furthermore, AGP was applied to assess the environmental performance of the three analytical methods, as shown in Fig. 4B.

### Modified GAPI greenness assessment method

The graphic depicts the Modified GAPI assessment’s total score, where the color scale around the pentagrams denotes the aggregate evaluation of the method. Likewise, Complex GAPI was recently modified to generate a total score of 96 (Fig. 4C), facilitating the assessment of analytical workflows that involve pre-analytical steps^65^.

### Greenness profile of Eco-scale

GAC provides a framework for incorporating environmental, health, and safety concerns into analytical practices. Techniques with Eco-scale scores above 75 are classified as environmentally favorable^66^, with higher scores (approaching 100) indicating superior greenness. The proposed method achieved an Eco-Scale score of 95, reflecting excellent environmental performance. Table 10 summarizes the penalty points assigned according to the Analytical Eco-Scale assessment.

**Table 10:** Penalty points (PPs) assigned to the developed method according to the analytical Eco-Scale.

Reagents	Analytical Eco- scale	PPs
	Methanol	5
	Ortho-phosphoric acid	0
Instruments	RP-HPLC	0
	**Total pp**	5
	**Eco –scale**	95

### Greenness profile of AGREE

AGREE serves as a fast assessment tool for quantitatively determining how well an analytical method complies with the twelve principles of green analytical chemistry. Higher scores are awarded to greener procedures, and the overall value is presented at the center of the circular diagram, as illustrated in Fig. 4D.

### Greenness profile of the BAGI blueness tool

The overall BAGI index is determined based on the relative weighting of individual scores and is used as a quantitative indicator of the practical feasibility of analytical procedures. Scores range between 25 and 100, with higher values reflecting improved practical performance. In the present work, the BAGI metric was applied to assess the blueness of the developed technique. The method obtained a score of 90, demonstrating high practicality, good production efficiency, automation potential, and economic viability, as shown in Fig. 4E.

## Conclusion

A validated green RP-HPLC method was successfully developed for the simultaneous determination of tedizolid phosphate (TP) and ciprofloxacin (CIP) in pharmaceutical formulations and human plasma. Rapid chromatographic separation was achieved within 4 min using an elevated flow rate, reducing analysis time while maintaining excellent analytical performance. Methanol was selected instead of acetonitrile because of its lower toxicity and more sustainable environmental profile without compromising linearity (R² > 0.999) or validation performance. Forced degradation studies performed on the pharmaceutical formulation demonstrated that the developed method was capable of monitoring the degradation behavior of TP and CIP under various stress conditions while maintaining chromatographic separation between the analyte peaks and the observed degradation products, supporting its potential as a stability-indicating method for quality control applications. The environmental performance of the method was comprehensively evaluated using NEMI, AGP, Modified GAPI, Eco-scale, AGREE, and BAGI metrics, confirming excellent greenness, practicality, and sustainability. Overall, the proposed method provides a rapid, reliable, environmentally sustainable, and stability-indicating analytical approach suitable for routine quality control and bioanalytical applications.

## Supplementary Information

Below is the link to the electronic supplementary material.

Supplementary Material 1

## Data Availability

The data supporting the findings of this study are available from the corresponding author upon reasonable request.

## Declarations

### Study limitations and future research plans

A limitation of the present study is that although extraction recovery and matrix effect were evaluated, these assessments were performed using a single human plasma lot, which does not fully account for inter-individual matrix variability. In addition, IS-normalized matrix factor determination and peak purity assessment during the forced degradation study were not performed, and long-term frozen storage stability was evaluated for only 7 days; therefore, extended frozen storage stability over longer storage periods was not investigated. Future studies will address these limitations to further strengthen the bioanalytical validation and regulatory applicability of the proposed method. Despite these limitations, the developed method demonstrated satisfactory selectivity, linearity, accuracy, precision, sensitivity, extraction recovery, matrix effect, and stability under the investigated conditions.
