# Supplementary material for: Green stability-indicating RP-HPLC method for simultaneous bioanalysis of tedizolid phosphate and ciprofloxacin in human plasma
Source: Sci Rep. 2026 Aug 13;16:25287. doi: 10.1038/s41598-026-65086-3 (PMC13473588; doi:10.1038/s41598-026-65086-3)
Supplement: Supplementary file 1 [file 41598_2026_65086_MOESM1_ESM.docx]

**Figures Captions:**

**Fig. S1.** Calibration curves for the analysis of **(A)** TP and **(B)** CIP in pure samples.

**Fig. S2.** Calibration curves for the analysis of **(A)** TP and **(B)** CIP in human plasma.

**Fig. S3.** Carry-over assessment: (A) highest plasma calibration standard (30 µg/mL TP); (B) subsequent blank plasma sample.

**Fig. S1.** Calibration curves for the analysis of **(A)** TP and **(B)** CIP in pure samples.

**Fig. S2.** Calibration curves for the analysis of **(A)** TP and **(B)** CIP in human plasma.

**Fig. S3.** Carry-over assessment: (A) highest plasma calibration standard (30 µg/mL TP); (B) subsequent blank plasma sample.

**Tables Captions:**

**Table S1. Back-calculated concentrations, %Bias, and %RSD for all tedizolid phosphate (TP) plasma calibration standards (n = 3).**

**Table S2.** Back-calculated concentrations, %Bias, and %RSD for all ciprofloxacin (CIP) plasma calibration standards (n = 3).

**Table S3. Dilution integrity results for tedizolid phosphate (TP) and ciprofloxacin (CIP) in spiked human plasma**.

**Table S4.** Extraction recovery of tedizolid phosphate (TP) and ciprofloxacin (CIP) from spiked human plasma at three quality control levels.

**Table S5.** Matrix effect evaluation of tedizolid phosphate (TP) and ciprofloxacin (CIP) in human plasma using the post-extraction addition method.

**Table S1. Back-calculated concentrations, %Bias, and %RSD for all tedizolid phosphate (TP) plasma calibration standards (n = 3)**.

| **Nominal concentration (µg/mL)** | **Back-calculated (Curve 1)** | **Back-calculated (Curve 2)** | **Back-calculated (Curve 3)** | **Mean (µg/mL)** | **Bias (%)** | **RSD (%)** |
| --- | --- | --- | --- | --- | --- | --- |
| 0.50 | 0.500 | 0.507 | 0.470 | 0.492 | −1.53 | 4.04 |
| 1.0 | 1.059 | 1.054 | 1.054 | 1.056 | +5.57 | 0.27 |
| 2.0 | 1.883 | 1.955 | 1.994 | 1.944 | −2.80 | 2.94 |
| 6.0 | 5.955 | 5.961 | 5.962 | 5.959 | −0.68 | 0.06 |
| 8.0 | 8.197 | 8.218 | 8.202 | 8.206 | +2.57 | 0.13 |
| 10.0 | 9.534 | 10.330 | 10.290 | 10.051 | +0.51 | 4.44 |
| 20.0 | 19.865 | 19.945 | 19.892 | 19.901 | −0.50 | 0.21 |
| 30.0 | 30.223 | 30.178 | 30.220 | 30.207 | +0.69 | 0.08 |

**Table S2.** Back-calculated concentrations, %Bias, and %RSD for all ciprofloxacin (CIP) plasma calibration standards (n = 3).

| **Nominal concentration (µg/mL)** | **Back-calculated (Curve 1)** | **Back-calculated (Curve 2)** | **Back-calculated (Curve 3)** | **Mean (µg/mL)** | **Bias (%)** | **RSD (%)** |
| --- | --- | --- | --- | --- | --- | --- |
| 0.5 | 0.50 | 0.508 | 0.480 | 0.496 | -0.80 | 2.87 |
| 1.0 | 1.01 | 1.04 | 1.073 | 1.041 | +4.10 | 3.03 |
| 2.0 | 1.925 | 1.779 | 2.127 | 1.944 | −2.79 | 9.03 |
| 4.0 | 3.867 | 3.855 | 3.603 | 3.775 | −5.62 | 4.04 |
| 6.0 | 5.988 | 6.400 | 5.707 | 6.032 | +0.54 | 5.77 |
| 8.0 | 8.020 | 7.744 | 8.378 | 8.047 | +0.58 | 3.95 |
| 10.0 | 10.043 | 10.01 | 10.447 | 10.167 | +1.67 | 2.42 |

**Table S3. Dilution integrity results for tedizolid phosphate (TP) and ciprofloxacin (CIP) in spiked human plasma**.

| **Analyte** | **Nominal concentration (µg/mL)** | **Dilution factor** | **Mean measured concentration (µg/mL)** | **Bias (%)** | **RSD (%)** |
| --- | --- | --- | --- | --- | --- |
| **TP** | 60.0 | 2 | 53.93 | −10.11 | 0.08 |
| **CIP** | 20.0 | 2 | 17.11 | −14.43 | 0.02 |

**Table S4.** Extraction recovery of tedizolid phosphate (TP) and ciprofloxacin (CIP) from spiked human plasma at three quality control levels.

| **Analyte** | **QC level** | **Concentration (µg/mL)** | **Pre-extraction response** | **Post-extraction response** | **Extraction recovery (%)** |
| --- | --- | --- | --- | --- | --- |
| **TP** | LQC | 0.5 | 0.00412 | 0.00420 | 98.10 |
|  | MQC | 2.0 | 0.06842 | 0.06980 | 98.02 |
|  | HQC | 10.0 | 0.53170 | 0.54400 | 97.74 |
| **CIP** | LQC | 0.5 | 0.05300 | 0.05435 | 97.52 |
|  | MQC | 2.0 | 0.35555 | 0.370324 | 96.01 |
|  | HQC | 10.0 | 1.55799 | 1.60056 | 97.34 |

**Table S5.** Matrix effect evaluation of tedizolid phosphate (TP) and ciprofloxacin (CIP) in human plasma using the post-extraction addition method.

| **Analyte** | **QC level** | **Concentration (µg/mL)** | **Post-extraction response** | **Neat standard response** | **Matrix Factor** |
| --- | --- | --- | --- | --- | --- |
| **TP** | LQC | 0.5 | 0.00420 | 0.00433 | 0.970 |
|  | MQC | 2.0 | 0.06980 | 0.06700 | 1.042 |
|  | HQC | 10.0 | 0.54400 | 0.51000 | 1.067 |
| **CIP** | LQC | 0.5 | 0.05435 | 0.05178 | 1.050 |
|  | MQC | 2.0 | 0.370324 | 0.37000 | 1.001 |
|  | HQC | 10.0 | 1.60056 | 1.59200 | 1.005 |
